# Return to the sea: *Sagaratrema* n. g. for the marine Liolopidae (Digenea: Diplostomida) with two new species parasitic in snakes from Sri Lanka

**DOI:** 10.1017/S0031182026101802

**Published:** 2026-04

**Authors:** Manage Lenin Indrajith De Silva, Erandi Pathirana, Jayanthe Rajapakse, Storm Martin

**Affiliations:** 1 Centre for Sustainable Aquatic Ecosystems, Harry Butler Institute, Murdoch University, Murdoch, WA, Australia; 2 Division of Parasitology, Department of Veterinary Pathobiology, Faculty of Veterinary Medicine and Animal Science, University of Peradeniya, Peradeniya, Sri Lanka; 3 Department of Fisheries and Ocean Sciences, Faculty of Fisheries and Ocean Sciences, Ocean University of Sri Lanka, Tangalle, Sri Lanka; 4 Department of Aquatic Bioresources, Faculty of Urban and Aquatic Bioresources, University of Sri Jayewardenepura, Nugegoda, Sri Lanka

**Keywords:** biogeography, Hydrophinae, *Hydrophis*, Indo-West Pacific, kraits, *Laticauda*, parasites, sea snakes, Trematoda

## Abstract

The Liolopidae Dollfus, 1934 is a small family of digenetic trematodes with sexual adults parasitic in aquatic reptiles and amphibians. Liolopids exploiting snakes are constrained to *Harmotrema* Nicoll, 1914, but the genus includes species with presumably freshwater life cycles parasitic in terrestrial snakes as well as species with presumably marine life cycles parasitic in viviparous sea snakes and amphibious kraits. We hypothesize that this ecological distinction implies substantial separation in evolutionary history and propose *Sagaratrema* De Silva, Pathirana & Martin n. g. to accommodate the liolopids in marine snakes. Three species are delineated through an integrated approach, from novel collections of viviparous sea snakes in Sri Lanka, *Sagaratrema rajapaksei* De Silva, Pathirana & Martin n. sp., *Sagaratrema rajakarunae* De Silva, Pathirana & Martin n. sp. and *Sagaratrema indicum* (Chattapadhyaya, 1970) n. comb. (= *H. indica*) originally reported from India. Three other species known from marine snakes are transferred from *Harmotrema* to the new genus: *S. laticaudae* (Yamaguti, 1933) n. comb. (designated as the type-species), *S. eugari* (Tubangui & Masilungan, 1936) n. comb. and *S. linguiforme* (Wang, 1987) n. comb. (= *H. linguiforme*). The 3 species from Sri Lanka are similarly genetically distinct in sympatry as each is relative to *S. laticaudae* from Japan. Following these proposals, *Harmotrema* is revised and rendered monotypic for the type-species *H. infecundum*. Morphologically, *Sagaratrema* is distinguished from *Harmotrema* and other liolopid genera by the arrangement of the excretory vesicles, distribution of the vitellarium and size and shape of the body.

## Graphical abstract

**Figure:**
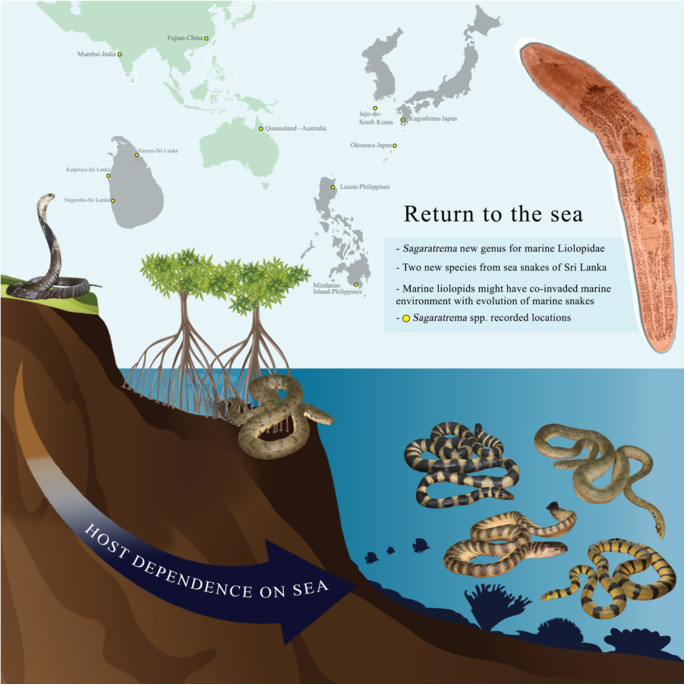

## Introduction

Digenean trematodes of the Liolopidae Dollfus, 1934 infect the gastrointestinal tract of reptiles and amphibians associated with aquatic habitats (Skrjabin, 1962; Brooks and Overstreet, 1978; Niewiadomska, 2002; Baba et al., 2011; Dutton et al., 2022, 2024). Higher classification for the Liolopidae remains problematic. The family unambiguously belongs to the Diplostomida but has variously been treated within each of the 3 diplostomidan superfamilies recognized at present, the Brachylaimoidea Joyeux & Foley, 1930, Diplostomoidea Poirier, 1886 and Schistosomatoidea Stiles & Hassall, 1898, as well in the previous concept of the Clinostomoidea Lühe, 1901 (Niewiadomska, 2002). Phylogenetic analyses have found the family to be genetically distinct and basal among the Diplostomida, resolving sister to either the Diplostomoidea or Schistosomatoidea (Baba et al., 2011; Hernández-Mena et al., 2017) or even sister to all other Diplostomida (Cutmore et al., 2023).

The Liolopidae currently comprises 6 genera, of which only *Harmotrema* Nicoll, 1914 includes parasites of snakes: *Liolope* Cohn, 1902 comprises the type-species *Liolope copulans* Cohn, 1902 from giant salamanders in East Asia as well as *L. dollfusi* Skrjabin, 1962 from a freshwater pelomedusid turtle in Gabon; *Helicotrema* Odhner 1912 comprises Neotropical species from iguanas, freshwater turtles and tortoises; *Dracovermis* Brooks and Overstreet, 1978 and *Ngubuvangandu* Dutton & Bullard, 2026 comprise species infecting crocodilians; and the monotypic *Paraharmotrema karinganiense* Dutton & Bullard, 2022, like *L. dollfusi*, infects freshwater pelomedusid turtles and is known from southeastern Africa (Chin et al., 1974; Brooks and Overstreet, 1978; Niewiadomska, 2002; Dutton et al., 2022, 2024, 2026). Recently, Numdi and Aisien (2021) reported an unidentified species of *Harmotrema* from the West African mud turtle *Pelusios castaneus* (Schweigger) (Pelomedusidae) in Nigeria, but that worm appears to be consistent with *P. karinganiense*.

Life cycle information for the Liolopidae is known from the type-species *L. copulans* in Japan (Ozaki and Okuda, 1951; Baba et al., 2011) and for *P. karinganiense* in southeastern Africa (Donough et al., 2025). For both species, natural infections have been reported from freshwater snails as first-intermediate hosts, and freshwater fishes as second-intermediate hosts. Presumably all liolopids require trophic transmission from vertebrate second-intermediate hosts (fishes or conceivably also amphibians), with the possible exception of *Helicotrema* spp. which are known from mostly herbivorous definitive hosts.

The only liolopid genus with species known from snakes, *Harmotrema*, currently comprises 5 recognized species. The type species *H. infecundum* Nicoll, 1914 is known only from Smith’s African water snake *Grayia smythii* Günther (Colubridae), from western Africa (Nicoll, 1914; Dollfus, 1950). Considering the habits of this semi-aquatic snake, transmission of *H. infecundum* presumably occurs in freshwater, likely involving fish or amphibian intermediate hosts. Conversely, 3 of the remaining 5 species are known only from marine elapid snakes: *H. laticaudae* Yamaguti, 1933 is known from the blue-lipped sea krait *Laticauda laticaudata* (Linnaeus) (Elapidae: Hydrophiinae: Laticaudini) in Japan (Yamaguti, 1933; Telford, 1967), the black-banded sea krait *L. semifasciata* (Reinwardt) in the Republic of Korea (Choe et al., 2020; Dutton et al., 2022), and the olive sea snake *Aipysurus laevis* Lacépède (Hydrophiinae: Hydrophinii) and the olive-headed sea snake *Hydrophis major* (Shaw) (Hydrophinii) in Australia (Brooks and Overstreet, 1978), whereas *Ha. indicum* Chattopadhyaya, 1970 emend. and *Ha. linguiforme* Wang, 1987 are known only from the beaked sea snake *Hy. schistosus* (Daudin) in India and the annulated sea snake *Hy. cyanocinctus* Daudin in Fujian, China, respectively (Chattopadhyaya, 1970; Wang, 1987). Presumably, these 3 species are transmitted to their definitive hosts via predation of marine fishes. Predicting the life cycle ecology for *Ha. eugari* Tubangui & Masilungan, 1936 is less straightforward. It is known in the Philippines from both a terrestrial snake, the Philippine cobra *Naja philippinensis* Taylor (Elapidae: Elapinae), and a snake which forages substantially in marine habitats, the Southeast Asian bockadam *Cerberus schneiderii* (Schlegel) (as *C. rynchops*) (Homalopsidae) (Tubangui and Masilungan, 1936; Tubangui, 1947; Fischthal and Kuntz, 1967; see also Murphy et al., 2012; Bernstein et al., 2024). Finally, *Ha. microrchis* Bhutta & Khan, 1975, from the gharial (Bhutta and Khan, 1975), is morphologically and ecologically inconsistent with the genus concept and has been considered a *species inquirenda* by Dutton et al. (2026).

This study reports on liolopids recovered during the first parasitological investigation of marine snakes in Sri Lanka. The specific identities of the recovered specimens were assessed using an integrated approach, and a new classification hypothesis is presented to reflect the significance of marine liolopids.

## Materials and methods

### Host and parasite collection

**Figure 1.**
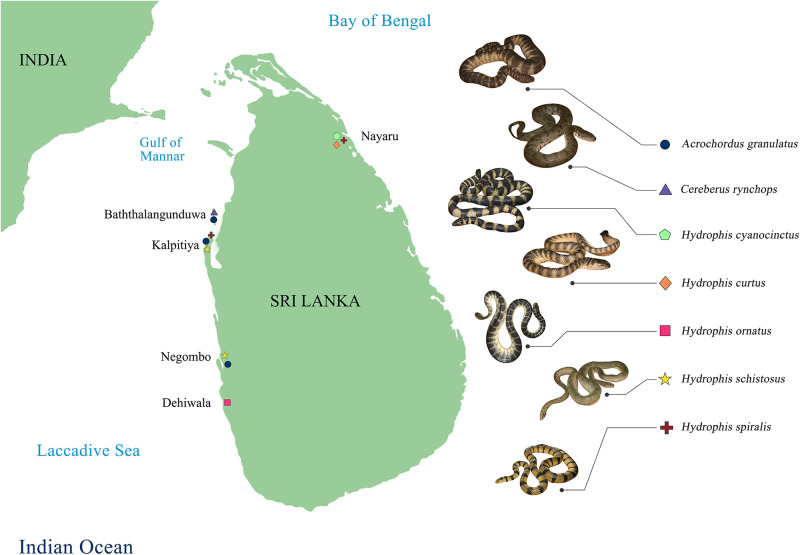
Landing locations for the marine snakes caught through fisheries bycatch in Sri Lanka and examined for parasites. Figure 1 long description.

Marine snakes from fishery bycatch were collected between August 2021 and August 2022 in Sri Lanka, from coastal waters of both the Gulf of Mannar, mostly from Portugal Bay, Northwestern Province and the Bay of Bengal, in the vicinity of Nayaru, Northern Province (see Figure 1). Examined snakes comprised 22 individuals belonging to 7 species: 7 little filesnakes *Acrochordus granulatus* (Schneider) (Acrochordidae) trawled in Portugal Bay, 2 landed at Kalpitiya, 4 landed at Baththalangunduwa and 1 landed at Negombo, 1 South Asian bockadam *Cerberus rynchops* (Schneider) (Homalopsidae) trawled in Portugal Bay and landed at Baththalangunduwa, 2 ornate reef sea snakes *Hydrophis ornatus* (Gray) from the Laccadive Sea landed at Dehiwala, 7 beaked sea snakes *Hy. schistosus* trawled in Portugal Bay and landed at Kalpitiya and Baththalangunduwa and from the Laccadive Sea landed at Negombo, 1 Shaw’s sea snake *Hy. curtus* (Shaw) and 2 annulated sea snakes *Hy. cyanocinctus* from the Bay of Bengal landed at Nayaru and 2 yellow sea snakes *Hy. spiralis* (Shaw), 1 each from the Gulf of Mannar landed at Kalpitiya and the Bay of Bengal landed at Nayaru. Fresh faecal samples were collected from *Hy. schistosus* by gently massaging/pressing the lower coelom towards the vent. Snakes and faecal samples were isolated in sealable bags, stored on ice and transported to the Division of Parasitology, Department of Veterinary Pathobiology, Faculty of Veterinary Medicine and Animal Science, University of Peradeniya, Sri Lanka. Snakes were examined and necropsied as detailed in Martin et al. (2023), and recovered trematodes were preserved in 70–95% ethanol or 10% formalin.

### Morphological study

Fresh parasite specimens and faecal samples were prepared as temporary mounted slides to observe gross morphology under dissecting and light microscopes. Permanent mounts of whole parasites were prepared, without flattening, from preserved specimens washed in distilled water, stained with Mayer’s haematoxylin and destained with diluted HCl solution (1%), neutralized with diluted NH_3_ solution (1%), dehydrated in a series of ethanol solutions of 50, 70, 90, 95 and twice in 100%, cleared in methyl salicylate, initially in a 50% solution with 100% ethanol and subsequently in a 100% solution, and finally mounted in Canada balsam. Some specimens preserved in formalin were stained in Van Cleave’s haematoxylin with several drops of Ehrlich’s haematoxylin.

Morphometric data were acquired through live feed using cellSens Standard v1.13, from an Olympus BX50 microscope equipped with Nomarski interference contrast and an Olympus DP71 digital camera with a UCMAD3 adaptor (Olympus Inc., Tokyo, Japan). Line drawings were drawn using a camera lucida from an Olympus BHA phase contrast microscope and subsequently digitized in Adobe Illustrator CC. Measurements are provided in micrometres (μm), with the range followed by mean and standard error in parentheses, length followed by width where applicable, and measurements for eggs averaged from 10 per specimen. Type specimens are lodged in the National Museum of Sri Lanka, Colombo (NMSL), with some paratypes deposited in the Crustacea and Worms collection of the Western Australian Museum, Perth (WAM).

### Molecular and phylogenetic study

Novel genetic sequence data were generated from hologenophores to prospect for genetic diversity and potential species richness. Sequences were generated for the cytochrome c oxidase subunit 1 mitochondrial marker (COI mtDNA), the noncoding second internal transcribed spacer unit of ribosomal DNA (ITS2 rDNA) and the large ribosomal subunit gene (28S rDNA). Specimens for molecular analysis were processed, and sequences generated and assembled, in accordance with the protocols in Martin et al. (2023). The partial COI region was amplified and sequenced using the primers Dig_cox1Fa (Wee et al., 2017) and Dig_cox1R (Wee et al., 2017), the ITS2 region using 3S (Morgan and Blair, 1995) and ITS2.2 (Cribb et al., 1998), and the partial 28S region using LSU5 (Littlewood, 1994) and 1500R (Snyder and Tkach, 2001).

For each of the 3 markers, the novel sequences were aligned with MUSCLE (Edgar, 2004) in MEGA 11 (Tamura et al., 2021) using default parameters and compared using simple distance matrices, unrooted neighbour-joining trees and manual inspection of alignments and corresponding chromatograms. The only relevant published sequence included in these alignments was GenBank OL413009 of Dutton et al. (2022), representative of 2 identical replicate 28S sequences generated from a collection of 5 specimens identified as *Harmotrema laticaudae* from a single *Laticauda semifasciata* at Ishigakai Island, Okinawa Prefecture, Japan, with corresponding morphological vouchers available in the Kyoto University Museum (KUZ Z3989) (M. Urabe, pers. comm.).

A phylogenetic hypothesis based on partial 28S rDNA was reconstructed for the Diplostomida via maximum likelihood analysis. The alignment was trimmed to 1276 nucleotides without masking ambiguously aligned regions and included 1 sequence each for all representatives of the Liolopidae with available data, as well as 1 sequence each per family with available data within the Brachylaimoidea, Diplostomatoidea and Schistosomatoidea (Table 1); a representative of the Transversotrematoidea (Plagiorchiida) was used as the outgroup. The analysis was performed using the implementation of RAxML v8.2.12 (Stamatakis, 2014) in the CIPRES portal (Miller et al., 2010). The analysis assumed the GTR + Γ model of nucleotide substitution and ran 1000 bootstrap pseudoreplicates as determined with the majority rule bootstopping criterion (autoMRE) (Pattengale et al., 2009).

**Table 1.** 28S rDNA sequence data representative of the taxonomic breadth of Diplostomida and used in the phylogenetic analysis Table 1 long description.

Taxon	Accession	Reference
**Liolopidae**		
*Liolope copulans* Cohn, 1902	AB551568	Baba et al. (2011)
*Dracovermis occidentalis* Brooks and Overstreet, 1978	PQ186364	Dutton et al. (2024)
*Paraharmotrema karinganiense* Dutton & Bullard in Dutton, Du Preez, Urabe & Bullard, 2022	OL413003	Dutton et al. (2022)
*Sagaratrema rajapaksei* De Silva, Pathirana & Martin, n. sp.	PX981822	*ex nobis*
*Sagaratrema rajakarunae* De Silva, Pathirana & Martin, n. sp.	PX981821	*ex nobis*
*Sagaratrema indicum* (Chattopadhyaya, 1971) De Silva, Pathirana & Martin	PX981820	*ex nobis*
**Brachylaimoidea**		
Brachylaimidae: *Brachylaima succini* Nakao, Sasaki, Tsukas & Waki, 2020	LC514748	Nakao et al. (2020)
Leucochloridiidae: Leucochloridium paradoxum Carus, 1835	LC466798	Nakao et al. (2019)
Brachylaimidae: *Zeylanurotrema spearei* Cribb & Barton, 1991	AY222170	Olson et al. (2003)
**Diplostomoidea**		
Diplostomidae: *Dungalabatrema kostadinovae* Achatz, Chermak & Tkach in Achatz, Chermak, Junkert & Tkach, 2022	OM569683	Achatz et al. (2022)
Diplostomidae: *Cotylurus cornutus* (Rudolphi, 1809) Szidat, 1928	PX457622	Stanevičiūtė et al. (2025)
Cyathocotylidae: *Braunina cordiformis* Wolf, 1903	KM258670	Fraija-Fernández et al. (2015)
**Schistosomatoidea**		
Carettacolidae: *Carettacola hawaiiensis* Dailey, Fast & Balazs, 1991	AY604709	Snyder (2004)
Hapalotrematidae: *Amphiorchis* sp. (Price, 1934)	KX987111	Cribb et al. (2017)
Schistosomatidae: *Austrobilharzia terrigalensis* Johnston, 1917	AY157249	Lockyer et al. (2003)
Atamatamidae: *Paratamatam iquitosiensis* Bullard & Roberts, 2019	MK775720	Bullard et al. (2019)
Spirorchiidae: *Spirorchis collinsi* Roberts & Bullard, 2016	KY091664	Roberts et al. (2016)
Unicaecidae: *Baracktrema obamai* Roberts, Platt & Bullard, 2016	KX061500	Roberts et al. (2016b)
Plattidae: *Platt snyderi* (Platt & Sharma, 2012) Roberts & Bullard, 2018	MF579528	Roberts et al. (2018)
Aporocotylidae: *Braya jexi* Nolan & Cribb, 2006	MZ264863	Yong et al. (2021)
Chimaerohemecidae: *Electrovermis zappum* Warren and Bullard, 2019	MN244242	Warren and Bullard (2019)
Elopicolidae: *Elopicola bristowi* Orélis-Ribeiro & Bullard	MH720952	Cutmore et al. (2018)
Clinostomidae: *Clinostomum marginatum* (Rudolphi, 1819) Braun, 1899	MF398323	Hernández-Mena et al. (2017)
Acipensericolidae: *Acipensericola petersoni* Bullard, Snyder, Jensen & Overstreet, 2008	KY243879	Orelis-Ribeiro et al. (2017)
Sanguinicolidae: *Pseudosanguinicola occidentalis*^a^ (Van Cleave & Mueller, 1932) Warren & Bullard, 2023	OQ715339	Warren et al. (2023)
**Transversotrematoidea**		
*Transversotrema tragorum* Hunter et al., 2012	GQ180853	Hunter et al. (2012)

a Registered name in GenBank as *Sanguinicola occidentalis.*

## Results

### Recovered material

Out of 22 marine snakes across 7 species examined, liolopid specimens were recovered from the small intestine of 1 of 1 *Hy. curtus*, 2 of 2 *Hy. cyanocinctus*, 6 of 7 *Hy. schistosus* and 2 of 2 *Hy. spiralis*. No liolopid infections were detected in 2 *Hy. ornatus*, 7 *A. granulatus* and 1 *C. rynchops*. The recovered liolopids were pale-white, attached firmly to the intestine epithelium, and were already dead despite necropsy of fresh snakes. Yellow-brown, oval eggs were recovered in faecal samples from *Hy. schistosus* and were presumed to be those of the liolopids as no other trematodes were detected in those particular individual snakes.

Partially digested and undigested prey fishes were identified in 5 species of marine snakes: *Hy. curtus* contained sprats (Clupeidae), eel tail catfishes (Plotosidae) and garden eels (Congridae); *Hy. cyanocinctus* contained gobies (Gobidae), *Hy. schistosus* contained rabbit fishes (Siganidae), croakers (Sciaenidae), terapons (Terapontidae) and puffers (Tetradontidae); and both *C. rynchops* and *A. granulatus* contained gobies (Gobiidae). Because these snakes were procured from bycatch, these prey fishes, especially those undigested, might have been predated from fishing nets.

### Species recognition

**Figure 2.**
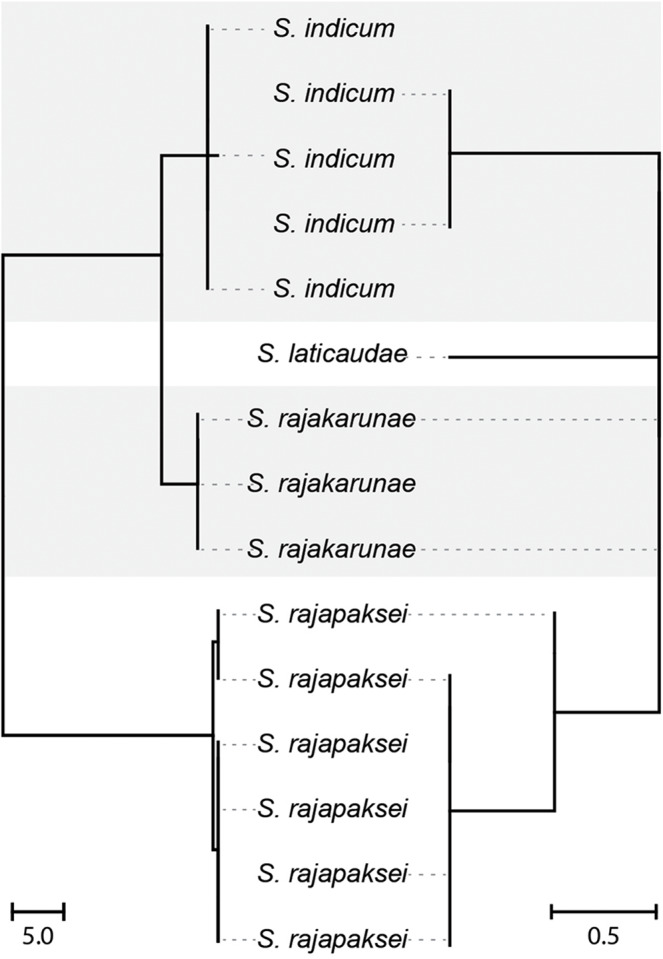
Unrooted neighbour-joining trees based on COI mtDNA (left) and 28S rDNA (right). Dotted horizontal lines indicate which data were generated from each specimen. The sequence representing *S. laticaudae* is GenBank OL413009 of Dutton et al. (2022); all other sequences are novel. The scale bars measure distance in number of base-positions. Figure 2 long description.

Following iterative investigation of morphology and genetic data, especially from hologenophore specimens, the novel collection from Sri Lanka is interpreted to represent 3 distinct species, all consistent with the current concept of *Harmotrema*. These species exhibit overlapping host and geographic ranges, including coinfections (Table 2). Generated molecular data comprised 11 partial COI mtDNA sequenced 485 base-positions in length, 9 ITS2 rDNA sequences 485 base-positions in length (including partial flanking 5.8S and 28S) and 8 partial 28S rDNA sequences 1,120 base-positions in length. These data were generated from 18 specimens from *Hy. cyanocinctus* caught in the Bay of Bengal and *Hy. schistosus* caught in the Gulf of Mannar. The ITS2 sequences were all identical but the 3 putative species were distinguished in both COI and 28S (Table 3). In COI, the 3 species corresponded to 3 haplogroups with intra-group variation of 0–1 base-positions (Figure 2). The 2 most similar haplogroups differed at 8–9 base-positions (2–2.2%), with a corresponding difference at 1 base-position in 28S. The third COI haplogroup differed to those 2 by 40–42 base positions (10.1–10.6%), with corresponding differences at 1 and 2 base-positions in 28S (some isolates also exhibited intraspecific polymorphisms, see Table 3). Importantly, these 3 genotypes in 28S, differing at 1–2 base-positions in sympatry in Sri Lanka, differed similarly by 1–2 base-positions from the available sequence identified as *Harmotrema laticaudae* from Japan (GenBank: OL413009; Dutton et al., 2022, see Figure 2). These 3 putative species are corroborated by morphological distinction, including relative to *H. laticaudae*. On the basis of morphology, one of the 3 recovered species is identified as *H. indicum* and the other 2 are considered species new to science.

**Table 2.** Host-parasite-locality combinations for novel material of *Sagaratrema* n. gen. spp. from *Hydrophis* spp. reported herein. Dots are positive combinations. Both instances of overlapping combinations included coinfections Table 2 long description.

Locality	Bay of Bengal			Gulf of Mannar	
Host	*H. curtus*	*H. cyanocinctus*	*H. spiralis*	*H. schistosus*	*H. spiralis*
Sample size	1	2	1	7	1
*S. indicum*	—	•	—	•	—
*S. rajapaksei*	•	—	•	•	•
*S. rajakarunae*	—	•	—	—	—

**Table 3.** Pairwise interspecific genetic differences between species of *Sagaratrema*, in number of base-positions, for partial 28S rDNA (1,120 base-positions) above the diagonal, and partial COI mtDNA (485 base-positions) below the diagonal. Intraspecific genetic variation is included for COI on the diagonal. For 28S, intragenomic polymorphic sites are counted as a difference of 0.5 if one of the 2 nucleotides present is common Table 3 long description.

	*S. indicum*	*S. rajapaksei*	*S. rajakarunae*	*S. laticaudae*
*Sagaratrema indicum*	0–1	1.5–2.5	1–1.5	2–2.5
*Sagaratrema rajapaksei*	41–42	0–1	0.5–1	1.5–2
*Sagaratrema rajakarunae*	8–9	40	0	1
*Sagaratrema laticaudae*	n/a	n/a	n/a	n/a

### Taxonomy

Descriptions for the 3 liolopid species recovered from sea snakes in Sri Lanka are provided below. Following consideration for morphology, biogeography and ecology, a new genus is proposed for liolopids from Indo-West Pacific marine snakes, and *Harmotrema* is revised.

Phylum Platyhelminthes Minot, 1876

Class Trematoda Rudolphi, 1808

Subclass Digenea Carus, 1863

Order Diplostomida Olson, Cribb, Tkach, Bray & Littlewood, 2003

Suborder Diplostomata Olson, Cribb, Tkach, Bray & Littlewood, 2003

Superfamily uncertain

Family Liolopidae Odhner, 1912

***Harmotrema*** Nicoll, 1914

Type-species: *Harmotrema infecundum* Nicoll, 1914

Revised diagnosis (marita): Body dorsoventrally flattened, elongate elliptical, broadest in mid third, gently tapered to bluntly pointed ends, 3–4.5 times longer than wide. Tegument unarmed. Suckers small, feeble; oral sucker slightly larger; ventral sucker in anterior fifth to third of body. Pharynx smaller than oral sucker. Oesophagus short but distinct. Intestine bifurcates in forebody; caeca blind, extend near to posterior end of body. Testes 2, tandem, medial (anterior testis may be dextro-submedial), with regular to irregular margins, well separated, in posterior two-fifths of body; post-testicular zone less than one-fifth of body length. Cirrus-sac large, oblique, spans intercaecal space, in hindbody and posterior half of body, substantially separated from ventral sucker, anteriad of gonads, contains bipartite seminal vesicle, pars prostatica and (presumed) eversible cirrus. Genital pore sinistro-submedial. Ovary dextro-submedial, globular with regular to slightly irregular margin, substantially smaller than and between testes. Mehlis gland large, similar in size to testes. Vitellarium follicular with 2 lateral fields; fields of follicles co-distributed with and strongly constrained about intestinal caeca, extend length of caeca in hindbody and well beyond ventral sucker into forebody but fall well short of intestinal bifurcation, may become confluent in post-testicular zone. Uterus short, intercaecal, between posterior testis and genital pore; oviduct extends posteriorly from ovary. Eggs very few (reported 2–5), very large, larger than ovary, 150–180 long. Excretory duct extends from terminal posterior excretory pore, bifurcates immediately into lateral branches near level of caeca termini, each lateral branch then bifurcates in midbody at the level of anterior testis to form loop with 1 extracaecal and 1 intercaecal branch extending anteriad and reuniting near to level of intestinal bifurcation. In intestine of African colubrid snakes frequenting freshwater.

Remarks: *Harmotrema* is here proposed to be reduced to monotypy. The revised diagnosis is based on the initial description of the type-species *H. infecundum* by Nicoll (1914) as well as an excellent supplemental description by Dollfus (1950) in which additional details were provided, most notably for the excretory system. The 5 species previously recognized in the genus are known only from snakes and are the only liolopids known from snakes. However, of these 5 species, the type-species *H. infecundum* is comparatively distinctive morphologically and ecologically; a new genus is proposed below for the remaining 4 species, as well as 2 new species, also proposed below. *Harmotrema infecundum* is known only from Smith’s African water snake *Grayia smythii* in Western Africa, a colubrid associated with freshwater systems, whereas all the remaining species are known from the Indo-West Pacific and mostly from marine snakes, specifically the viviparous sea snakes (Elapidae: Hydrophinae: Hydrophinii) and the amphibious sea kraits (Hydrophinae: Laticaudini: *Laticauda* spp.), with 1 species, *H. eugari*, known from the South Asian bockadam *Cerberus schneiderii* (Homalopsidae) (as *C. rynchops*, see Murphy et al., 2012) as well as a terrestrial snake, the Philippine cobra *Naja philippinensis* (Elapidae: Elapinae). Relative to these Indo-West Pacific species, in *H. infecundum* the loops of the lateral excretory ducts reach posteriorly only to the midbody near to the level of the anterior testis vs span the length of the caeca, the body is smaller and less elongate, the eggs are fewer and larger, the testes more posterior and irregular vs smooth, the forebody is longer (i.e. the ventral sucker is situated more posteriorly), the vitelline zone is longer (the follicles extend significantly into the forebody) and the post-testicular zone is shorter.

***Sagaratrema*** **De Silva, Pathirana & Martin n. g.**

Type-species: *S. laticaudae* (Yamaguti, 1933) n. comb. (= *Harmotrema laticaudae*)

Other recognized species: *S. eugari* (Tubangui and Masilungan, 1936) n. comb. (= *H. eugari*), *S. indicum* (Chattopadhyaya, 1970) n. comb. (= *H. indica, H. indicum*), *S. linguiforme* (Wang, 1987) n. comb. (= *H. linguiforme*), *S. rajapaksei* De Silva, Pathirana & Martin n. sp., *S. rajakarunae* De Silva, Pathirana & Martin n. sp. (comparative measurement data in Table 4)

**Table 4.** Morphometric data from reports of *Sagaratrema* spp Table 4 long description.

Species	*S. laticaudae*	*S. laticaudae*	*S. eugari*	*S. linguiforme*	*S. indicum*	*S. indicum*	*S. rajapaksei*	*S. rajakarunae*
Reference	Yamaguti (1933)	Choe et al. (2020)	Tubangui and Masilungan (1936)	Wang (1987)	Chattopadhyaya, 1970	*ex nobis*	*ex nobis*	*ex nobis*
Location	Japan	Korea	Philippines	China	India	Sri Lanka	Sri Lanka	Sri Lanka
Specimens	3		—	3	—	12	10	10
BL	5700–6900	6022–6818	1900–4000	8000–8800	5070–6020	5299–7594	5179–8430	4194–7611
BW	1000–2000	745–1136	500–700	1680–1760	910–1400	524–1006	565–1162	511–824
OSL	75–100	82–123	70–90	128–160	90–110	39–102	63–115	31–111
OSW	110–120	109–141	60–90	128–160	90–110	35–128	47–129	39–88
PhL	90–110	100–109	60–70	112–128	50–80	58–105	71–108	52–102
PhW	—	100–127	60–70	128–144	60–90	76–113	79–134	50–101
VSL	110–130	127–191	90–120	240–242	110–160	122–168	117–222	103–183
VSW	110–175	127–191	90–120	240–272	110–160	110–165	110–224	97–175
FbL	975*	1209–1309	650*	1600*	900*	867–1267	800–1383	748–1263
CSL	800	600–664	280–380	800	550	338–576	347–574	305–526
CSW	430	318–382	130–190	—	—	160–276	171–315	187–301
ATL	500–550	382–509	170–230	228–400	290–360	269–456	289–418	194–301
ATW	440–600	382–436	130–170	460–480	270–340	183–323	233–416	205–405
PTL	600–700	473–573	190–280	400–420	380–390	305–459	322–541	179–307
PTW	450–560	350–382	100–150	480–482	220–280	160–291	182–378	110–194
AT to PT	—	0–745	—	—	—	388–704	311–953	381–766
OvL	160–250	136–209	120–170	480–560	120–360	89–157	118–246	110–194
OvW	230–310	164–281	100–150	620–640	110–200	82–159	101–223	91–198
EL	123–129	111–125	108–115	105–108	109–148	101–124	104–123	103–130
EW	75–78	59–77	69–77	68–70	62–73	63–80	63–79	59–76
No. eggs	14–40*	31*	12*	10*	12*	12–23	25–47	5–17
Post-TZL	1300*	1380*	1280*	2800*	2000*	1388–2229	1421–3090	1314–2836
Post-TZL/BL	20%*	21%*	43%*	33%*	36%*	23–39%	26–41%	31–39%
FbL/BL	15%*	20%*	22%*	19%*	16%*	13–21%	13–17%	14–21%
CS/BL	12%*	10%*	8%*	9%*	9%*	6–9%	5–9%	6–8%
PTL/BL	10%*	8%*	8%*	5%*	7%*	5–8%	5–7%	5–6%
PTW/BL	8%*	6%*	4%*	6%*	5%*	3–5%	2–5%	3–4%

Abbreviations: L, length; W, width; B, body; OS, oral sucker; Ph, pharynx; VS, ventral sucker; Fb, forebody; CS, cirrus-sac; AT, anterior testis; PT, posterior testis; Ov, ovary; E, egg; Post-TZ, post-testis zone. An asterisk indicates measurement not provided but taken from illustration. A dash indicates data unavailable. Units in micrometres.

Etymology: From Sinhala සාගර (sāgara, =ocean) and *trema* from trematode, because we hypothesize that at least some species of the genus have marine life cycles, and in honour of the Ocean University of Sri Lanka, where M.L.I. De Silva and E. Pathirana first conceived of this research. The genus is neuter.

Diagnosis (marita): Body dorsoventrally flattened, elongate linguiform, gently tapered to bluntly pointed ends, 4.5–7.5 times longer than wide. Tegument may have minute spines/scales. Suckers small, feeble; oral sucker weakly muscular, terminal to subterminal, smaller than ventral sucker; ventral sucker reduced to circular, flattened disc with central concave depression surrounded by flimsy skirt of muscle, in anterior fifth to quarter of body. Pharynx small, similar in size to oral sucker. Oesophagus short but distinct. Intestine bifurcates in forebody; caeca blind, narrow, extend near to posterior extremity of body. Testes 2, tandem, medial, ellipsoidal, smooth, well separated, in mid hindbody; post-testicular zone substantial, one-fifth to one-third body length. Cirrus-sac large, oblique, spans intercaecal space, in hindbody but anterior half of body, substantially separated from ventral sucker, anterior to gonads, contains bipartite seminal vesicle, pars prostatica and spined, eversible cirrus. Genital pore sinistro-submedial. Ovary medial to dextro-submedial, spherical, smooth, smaller than and between testes. Vitellarium follicular with 2 lateral fields; vitelline reservoir small, posterior to ovary; fields of follicles co-distributed with intestinal caeca, restricted to hindbody (or reach level of ventral sucker or just anteriad), confluent, fill much of intercaecal space in post-testicular zone and between ventral sucker and cirrus sac, mostly excluded from intercaecal space between cirrus-sac and posterior testis. Uterus short, intercaecal, between posterior testis and genital pore, passes ventral to anterior testis; oviduct extends posteriorly from ovary; uterine seminal receptacle in proximal coils; metraterm well-developed. Eggs operculate, many (approximately 20–50), large, smaller than ovary, 105–130 long. Excretory pore subterminal. Excretory vessel divided into 4 parallel vessels; primary vessel bifurcates immediately anterior to excretory pore into paired lateral vessels; lateral vessels each bifurcate near termini of caeca into 1 extracaecal and 1 intercaecal vessel; extracaecal and intercaecal vessels run length of intestinal caeca, reunite in forebody at level of intestinal bifurcation or oesophagus; lateral vessels reconnect via narrow, transverse loop dorsal to pharynx. In intestine of Indo-West Pacific and mostly marine snakes.

Differential diagnosis: Body elongate linguiform with length 4.5–7.5 times width vs ovoid, pyriform or linguiform with length <4.5 width in *Liolope, Dracovermis, Ngubuvangandu*. Ventral sucker feeble vs strongly muscular in *Liolope*. Testes entire vs deeply lobed in *Paraharmotrema*. Post-testicular zone one-fifth to one-third body vs less than one-fifth in *Harmotrema*, greater than one-half in *Helicotrema*. Cirrus-sac separated from ventral sucker vs immediately posteriad in *Liolope*. Vitelline fields confluent vs separate in *Harmotrema, Paraharmotrema*; restricted to hindbody vs enter forebody substantially in *Harmotrema, Liolope, Ngubuvangandu*; extend anteriorly near to level of ventral sucker vs at most to level of cirrus-sac in *Helicotrema*. Eggs large (105–130 µm long) but smaller than in *Harmotrema* (150–186 µm long), *Paraharmotrema* (130–160 µm long), many (approximately 5–47) vs fewer in *Harmotrema* (2–5), *Dracovermis* (1–7), *Ngubuvangandu* (< 15), *Liolope* (up to 23). Excretory vessels with loops spanning length of intestinal caeca vs anterior half in *Harmotrema, Paraharmotrema*.

**Figure 3.**
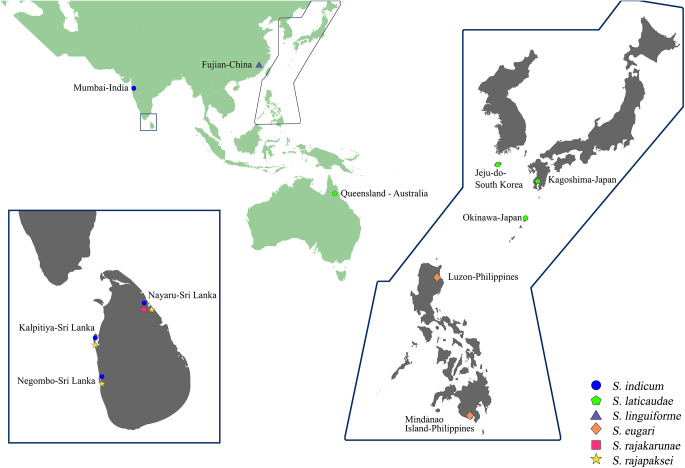
Known geographic range for recognized species of *Sagaratrema*. Figure 3 long description.

Remarks: *Sagaratrema* is proposed for the liolopids known from Indo-West Pacific and marine snakes; the known geographic range for the recognized species is depicted in Figure 3. These species are united by, and distinguished from, all other liolopids by the combination of the arrangement of the excretory system and the distribution of the vitellarium. In mounted specimens, the ventral sucker sometimes appears muscular with sucker walls folded inwards due to the cover slip pressure. Chattopadhyaya (1970) briefly described minute tegumental spines in *S. indicum*. We did not detect tegument spines or scales in any of the Sri Lankan specimens when examined under light microscope; imaging with scanning electron microscopy is warranted to understand the nature of the tegument.

Key to species of *Sagaratrema*

- Post-testicular zone one-fifth to one-quarter of body length; cirrus-sac occupies > one-tenth body length …….…………………………………………………............ *S. laticaudae*
- Post-testicular zone approximately one-third body length; cirrus-sac occupies < one-tenth body length ………….... 2
- Vitelline follicles constrained laterally, mostly restricted to intercaecal zone (especially anterior to cirrus-sac and posterior to testes)………………………………………….... 3
- Vitelline follicles extend laterally beyond caeca near to body margins………………………………………………........ 4
- Body approximately 2–4 mm long, vitelline follicles do not reach level of ventral sucker ………………………………………………………………….......... *S. eugari*
- Body approximately 5–6.5 mm long, vitelline follicles always reach level of ventral sucker ……………………………………………….……………….................. *S. indicum*
- Posterior testis wider than longer; egg length <110µm…………………………………………………………………………………………................ *S. linguiforme*
- Posterior testis longer than wide, egg length usually >110 µm…………………………………………............ 5
- Eggs number >20, vitelline follicles reach level of ventral sucker ………………………………………... *S. rajapaksei*
- Eggs number <20, vitelline follicles do not reach level of ventral sucker.....................................................*S. rajakarunae*

***Sagaratrema indicum*** (**Chattopadhyaya**, 1970) **n. comb**.

Synonyms: *Harmotrema indicum* Chattopadhyaya, 1970; *Harmotrema indica* Chattopadhyaya, 1970

Type-host: *Hydrophis schistosus* (Daudin) (as *Enhydrina schistosa*).

Type-locality: Off the coast of Mumbai (as Bombay), India.

Other previous reports: none.

Novel hosts, localities: *Hydrophis schistosus* from Portugal Bay and the Laccadive Sea landed at Kalpitiya, Baththalangunduwa and Negombo, Sri Lanka; *Hydrophis cyanocinctus* Daudin from the Bay of Bengal, landed at Nayaru, Sri Lanka.

Material examined: 27 novel specimens, comprising 23 from *Hy. schistosus* and 4 from 2 *Hy. cyanocinctus*.

Material deposited: 10 voucher specimens deposited, 4 in NMSL and 6 in WAM, V13583–88 including 4 hologenophores (WAM V13585–88).

Genetic sequences: COI (partial): 3 sequences varying at 0–1 base-positions with 2 deposited, PX979754 and PX979755 representative of 2 and 1 replicates, respectively; ITS2 (with flanking 5.8S and 28S): 3 identical sequences with 1 deposited, PX981823; 28S (partial): 2 identical replicates with 1 deposited, PX981820. All sequences from *Hy. schistosus*.

**Figure 4.**
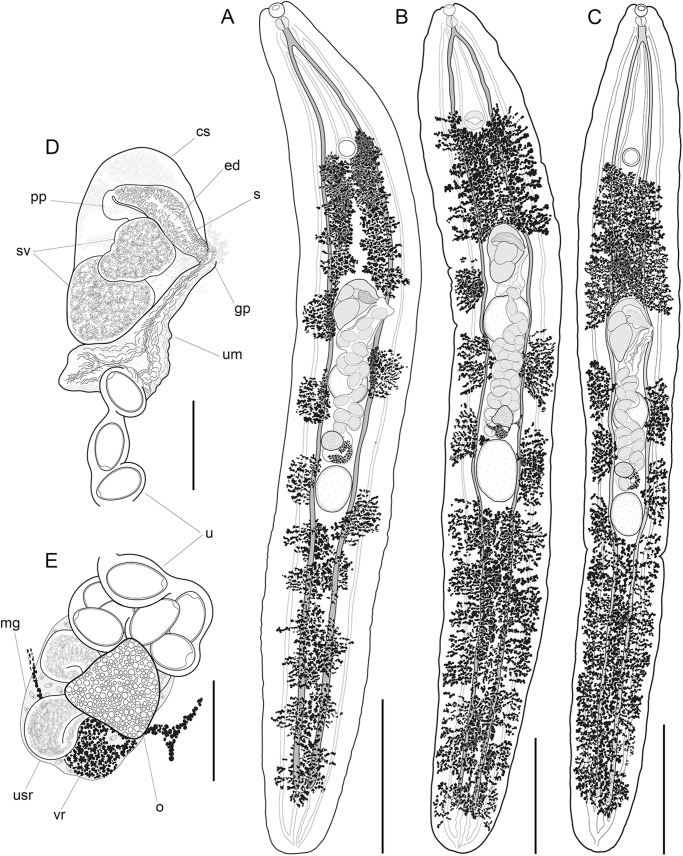
Species of *Sagaratrema* recovered from marine snakes of Sri Lanka. All illustrations from ventral perspective. (A) *Sagaratrema indicum* (Chattopadhyaya, 1970) n. comb., voucher from *Hydrophis cyanocinctus*. (B) *Sagaratrema rajapaksei* n. sp., holotype, from *Hydrophis curtus*. (C) *Sagaratrema rajakarunae* n. sp., holotype, from *Hydrophis cyanocinctus*. (D) *Sagaratrema rajapaksei* n. sp., holotype, terminal genitalia. (E) *Sagaratrema rajapaksei* n. sp., holotype, ovarian complex. Terminal genitalia and ovarian complex similar among species. Abbreviations: cs, cirrus-sac; ed, ejaculatory duct; gp, genital pore; mg, Mehlis’ gland; o, ovary; pp, pars prostatica; s, spines; sv, seminal vesicle; u, uterus with eggs; um, uterine metraterm; usr, uterine seminal receptacle; vr, vitelline reservoir. Scale bars: A–C, 1 cm; D–E, 200 µm. Figure 4 long description.

Supplementary description (Figure 4A): Based on 12 gravid specimens. Body dorsoventrally flattened, elongate linguiform, broadest in region between ventral sucker and anterior testis, gently tapered to bluntly pointed ends, 5299–7594 (6016 ± 728) × 524–1006 (805 ± 141), length 6.3–11 (7.4 ± 1.5) times width. Forebody 867–1267 (1061 ± 101) or 13–20 (18 ± 2)% of body length. Tegument seemingly unarmed. Oral sucker small, circular, terminal to subterminal, 39–102 (71 ± 21) × 35–128 (101 ± 30). Ventral sucker small, feeble, flattened disc with flimsy muscular walls, 122–168 (146 ± 16.9) × 110–165 (129 ± 18). Pharynx muscular, slightly smaller than oral sucker, 58–105 (73 ± 15) × 76–113 (91 ± 15). Prepharynx indistinct. Oesophagus short. Intestine bifurcated; caeca narrow, mostly straight, terminate near posterior end of body.

Testes 2, tandem, medial, smooth, longer than wide, span intercaecal space, well separated; anterior testis subspherical, 269–456 (356 ± 55) × 183–323 (272 ± 40), separated from cirrus-sac by short distance 196–441 (279 ± 66) or 4–6 (5 ± 0.67)% of body length; posterior testis elongate ellipsoidal, often with transverse notch so as to appear weakly bilobed, 305–459 (375 ± 43) × 160–291 (251 ± 39), separated from anterior testis by 388–704 (558 ± 121) or 7–9 (9 ± 1.5)% of body length; post-testicular zone 1388–2229 (1784 ± 244) or 23–39 (30 ± 5)% of body length. Cirrus-sac large, slightly oblique, 338–576 (448 ± 66) × 160–276 (217 ± 31.7), occupies 6–9 (7 ± 0.9)% of body length, situated 1951–3360 (2235 ± 376) or 34–44 (38 ± 3)% of body length from anterior body extremity, situated 734–1925 (1047 ± 332) or 14–25 (17 ± 3.93)% of body length from ventral sucker. Seminal vesicle internal, bipartite with anterior part slightly smaller. Pars prostatica short, simple; prostatic cells fill space within cirrus-sac around seminal vesicle and cirrus. Cirrus spined. Common genital atrium indistinct. Genital pore sinistro-submedial, intercaecal.

Ovary spherical, dextro-submedial, between testes, closer to posterior testis, 89–157 (120 ± 20) × 82–159 (123 ± 22). Vitelline reservoir postero-dorsal to ovary. Vitelline follicles small, irregularly rounded, co-distributed throughout hindbody along intestinal caeca dorsally and ventrally in 2 lateral, confluent fields, extend anteriorly to level of ventral sucker or sometimes just anterior, laterally more extensive dorsally than ventrally, predominately confined to intercaecal space in zone between ventral sucker and cirrus-sac and in post-testicular zone, excluded from intercaecal space and in asymmetrical patches about caeca in zone between cirrus-sac and posterior testis, in some specimens limited to 1 or 2 patches. Uterus short, intercaecal, constrained between posterior testis and genital pore, ventral to anterior testis; uterine seminal receptacle prominent in proximal part of uterus; metraterm muscular, well developed. Eggs oval, 12–23 (*n* = 17) in number, 101–124 (110 ± 6.10) × 63–80 (71 ± 5). Excretory pore subterminal. Excretory vessel lateral loops span length of caeca.

Remarks: The novel material is consistent with *H. indicum* as described by Chattopadhyaya (1970) and is reported here from the same host species, among others, in proximity to the type-locality, at least relative to reports of other known species recognized here in *Sagaratrema*. Morphologically, the species is recognized by its laterally constrained vitelline distribution. Chattopadhyaya (1970) also reported a transverse notch on the posterior testis, which we observed in some but not all specimens, but also in some specimens of other species. Chattopadhyaya (1970) differentiated *S. indicum*, in part, for the presence of minute, backward facing tegumental spines. We did not observe spines in our specimens for any species of *Sagaratrema* and so do not rely on these as a distinguishing characteristic for *S. indicum*.

*Sagaratrema indicum* closely resembles *S. eugari* in which the vitelline follicles are similar by being mostly constrained to the intercaecal space. Chattopadhyaya (1970) distinguished the 2 by the anterior extent of the vitelline follicles, extending to the level of the ventral sucker in *S. indicum* vs falling short by a small distance in *S. eugari*. It is not clear for how many specimens Tubangui and Masilungan (1936) based their description of *S. eugari*. Nevertheless, in the novel material identifies as *S. indicum*, the vitelline follicles reached to the level of the ventral sucker or just beyond anteriorly in all specimens. Furthermore, *S. indicum* attains greater overall size (length 5070–6020 in the original material and 5385–6349 in the novel material vs 1900–4000 in *S. eugari*) and appears to have larger eggs (length 109–148 and 101–124 vs 108–115).

*Sagaratrema indicum* reliably differs from *S. laticaudae* by having a relatively longer post-testicular zone, smaller testes, a smaller cirrus-sack and fewer vitelline follicles predominantly confined to the intercaecal space. *Sagaratrema indicum* can be differentiated from *S. linguiforme*, which attains much larger size (8000–8800 long), has a laterally more extensive vitelline distribution and a subspherical to triangular posterior testis (vs elongate ellipsoidal).

***Sagaratrema rajapaksei***, **De SilvaPathirana & Martin n. sp.**

Type-host: *Hydrophis curtus* (Shaw).

Type-locality: Bay of Bengal, landed at Nayaru, Northern Province, Sri Lanka.

Other hosts and localities: *Hydrophis spiralis* (Shaw) from the Bay of Bengal landed at Nayaru, and *Hydrophis schistosus* (Daudin) and *Hydrophis spiralis* from the Gulf of Mannar including Portugal Bay and landed at Kalpitiya, Baththalangunduwa and Negombo, Sri Lanka.

Site of infection: Intestine.

Material examined: 17 specimens, comprising 14 from *Hy. schistosus*, 2 from *Hy. spiralis* and 1 from *Hy. curtus*.

Material deposited: 11 specimens deposited: holotype and 2 paratypes in NMSL, 8 paratypes in WAM (13568–75) including 4 hologenophores (WAM 13572–75).

Genetic sequences: COI (partial): 5 sequences differing at 0–1 base-positions with 2 deposited, PX979756 and PX979757 representative of 3 and 2 replicates, respectively; ITS2 (with flanking 5.8S and 28S): 5 identical sequences with 1 deposited, PX981824; 28S (partial): 4 identical replicates with 1 deposited, PX981822. All sequences from *Hy. schistosus*.

Zoobank LSID: https://zoobank.org/urn:lsid:zoobank.org:act:0B18AFE0-A16F-4170-A333-F44808FA6B9A

Etymology: In honour of the esteemed parasitologist, R.P.V.J Rajapakse of the University of Peradeniya, Sri Lanka, in recognition of his significant contributions to the field of parasitology.

Description: (Figures 4 & E) based on 10 gravid specimens. Body dorsoventrally flattened, elongate linguiform, broadest in region between ventral sucker and anterior testis, gently tapered to bluntly pointed ends, 5350–8430 (7114 ± 1088) × 565–1162 (815 ± 226) or 6–15 (9 ± 4) times width. Forebody 886–1383 (1094 ± 159.8) or 13–17 (15 ± 1.3)% of body length. Tegument seemingly unarmed. Oral sucker small, transverse ellipsoidal, terminal or subterminal 63–115 (89 ± 19) × 47–129 (97 ± 31). Ventral sucker small, feeble, flattened disc with flimsy muscular walls, 117–222 (164 ± 31) × 110–224 (155 ± 38). Pharynx muscular, slightly smaller than oral sucker, 71–108 (91 ± 13) × 79–134 (103 ± 17.2). Prepharynx indistinct. Oesophagus short. Intestine bifurcates below oesophagus; caeca narrow, mostly straight, terminate near posterior end of body.

Testes 2, tandem, medial, smooth, longer than wide, span intercaecal space, well separated; anterior testis subspherical, 289–418 (365 ± 49) × 233–416 (301 ± 63.7), separated from cirrus-sac by short distance 145–783 (432 ± 195) or 2–9 (6 ± 2.78)% of body length; posterior testis elongate ellipsoidal, often with transverse notch so as to appear weakly bilobed, 322–541 (426 ± 76.2) × 182–378 (285 ± 60), separated from anterior testis by 311–953 (655 ± 189.4) or 10–13 (11 ± 0.9)% of body length; post-testicular zone 1421–3090 (2377 ± 563.5) or 26–41 (33 ± 6.4)% of body length. Cirrus-sac large, slightly oblique, 347–574 (497 ± 69.8) × 171–315 (244 ± 54.6), occupies 5–9 (6 ± 1.4)% of body length, situated 1753–3258 (2320 ± 475) or 27–39 (32 ± 4.6)% of body length from anterior body extremity, situated 647–1864 (1102 ± 350) or 10–22 (15 ± 4.2)% of body length from ventral sucker. Seminal vesicle bipartite, enclosed with cirrus. Anterior part of seminal vesicle small, overlapping with large posterior vesicle. Pars prostatica short, simple; prostatic cells fill space within cirrus-sac around seminal vesicle and cirrus. Cirrus spined and club shaped. Common genital atrium indistinct. Genital pore sinistro-submedial, intercaecal.

Ovary spherical, dextro-submedial, between testes, closer to posterior testis, 118–246 (169 ± 37.8) × 101–223 (159 ± 46.3). Vitelline follicles small, irregularly rounded, co-distributed throughout hindbody along intestinal caeca dorsally and ventrally in 2 lateral, confluent fields, extend anteriorly to level of ventral sucker or sometimes just anterior, laterally more extensive dorsally than ventrally, extend laterally beyond intestinal caeca near to body margins, mostly excluded from intercaecal space and in 3–5 asymmetrical patches about caeca in zone between cirrus-sac and posterior testis. Uterus short, intercaecal, constrained between posterior testis and genital pore, ventral to anterior testis; uterine seminal receptacle prominent in proximal part of uterus; metraterm muscular, well developed. Eggs oval, 23–47 (*n* = 17) in number, 104–123 (115 ± 5.8) × 63–79 (69 ± 4.7). Excretory pore subterminal. Excretory vessel lateral loops span length of caeca.

Remarks: *Sagaratrema rajapaksei* is most similar to *S. indicum*, *and is similarly* distinguished from *S. laticaudae* by a relatively longer post-testicular zone, smaller testes and smaller cirrus-sac, from *S. eugari* by a larger body and eggs, and relatively larger testes, and from *S. linguiforme* by the shape of the posterior testis, larger eggs and a smaller body. *Sagaratrema rajapaksei* is reliably distinguished from *S. indicum* by the more extensive lateral distribution of the vitelline follicles. In *S. indicum*, the dorsal and ventral distributions of the vitelline follicles are mostly restricted to the intercaecal space in the post-testicular zone, though extend further laterally to the extracaecal excretory vessels in the zone between the ventral sucker and cirrus-sac. In *S. rajapaksei,* the dorsal follicles extend laterally more substantially beyond the caeca and extracaecal excretory vessels to almost fill the available space. This distinction also applies to the vitelline follicles in the region of the gonads and cirrus-sac, but less obviously so because, in both species, and indeed in all species of the genus, the vitelline follicles are mostly excluded from the intercaecal space in this mid-body region where their distribution is typically and variously disrupted.

***Sagaratrema rajakarunae*** **De Silva, Pathiran & Martin n. sp.**

Type-host: *Hydrophis cyanocinctus* Daudin.

Type-locality: Bay of Bengal, landed at Nayaru, Northern Province, Sri Lanka.

Other hosts and localities: none.

Site of infection: Intestine.

Material examined: 17 novel specimens

Material deposited: 10 specimens deposited: holotype and 2 paratypes in NMSL, 7 paratypes in WAM (V13576–13582) including 2 hologenophores (WAM 13581– 82).

Genetic sequences: COI (partial): 3 identical sequences with 1 deposited, PX979758; ITS2 (with flanking 5.8S and 28S): 2 identical sequences with 1 deposited, PX981825; 28S (partial): 2 identical replicates with 1 deposited, PX981821.

Zoobank LSID: https://zoobank.org/urn:lsid:zoobank.org:act:150F0D94-6A51-4622-86FF-BF01F7323DC8

Etymology: In honour of the esteemed parasitologist, R.S. Rajakaruna of the University of Peradeniya, Sri Lanka, in recognition of her significant contributions to the field of parasitology.

Description: (Figure 4C): Based on 10 gravid specimens. Body dorsoventrally flattened, elongate linguiform, gently tapered to bluntly pointed ends, 4194–7611 (6202 ± 1373) × 511–824 (686 ± 100) or 7–11 (9 ± 1.21) times width. Forebody 748–1263 (1039 ± 182) or 14–21 (16 ± 2.4)% of body length. Tegument seemingly unarmed. Oral sucker small, circular, terminal to subterminal 31–111 (71 ± 32) × 39–88 (67 ± 18). Ventral sucker small, feeble, flattened disc with flimsy muscular walls, 103–183 (147 ± 32) × 97–175 (134 ± 23). Pharynx muscular, slightly smaller than oral sucker, 52–102 (72 ± 16.3) × 50–101 (78 ± 15.3). Prepharynx indistinct. Oesophagus short. Intestine bifurcates level of oesophagus; caeca narrow, mostly straight, terminate near posterior end of body.

Testes 2, tandem, medial, smooth, longer than wide, span intercaecal space, well separated; anterior testis subspherical, 194–357 (295 ± 33.5) × 194–301 (244 ± 33.5), separated from cirrus-sac by short distance 172–558 (321 ± 117.4) or 3–7 (5 ± 1.4)% of body length; posterior testis elongate ellipsoidal, often with transverse notch so as to appear weakly bilobed, 205–405 (326 ± 60) × 179–307 (235 ± 46.5), separated from anterior testis by 381–766 (598 ± 132) or 7–12 (9 ± 1.7)% of body length; post-testicular zone 1314–2836 (2209 ± 568) or 31–39 (35 ± 2.7)% of body length. Cirrus-sac large, slightly oblique, 305–526 (432 ± 62.4) × 187–301 (252 ± 30.9), occupies 6–8 (7 ± 0.8)% of body length, situated 1613–2539 (2149 ± 362.1) or 28–38 (33 ± 3.7)% of body length from anterior body extremity and 629–1272 (962 ± 192.2) or 11–17 (15 ± 2.5)% of body length from ventral sucker. Seminal vesicle internal, bipartite, enclosed with cirrus with anterior part slightly smaller. Pars prostatica short, simple. Cirrus spined. Common genital atrium indistinct. Genital pore sinistro-submedial, intercaecal.

Ovary spherical, dextro-submedial, between testis, closer to posterior testis, 110–194 (152 ± 30.7) × 91–198 (147 ± 34). Vitelline reservoir postero-dorsal to ovary. Vitelline follicles small, irregularly rounded, co-distributed throughout hindbody along intestinal caeca in 2 lateral, confluent fields, extend anteriorly near to but separated from posterior margin of ventral sucker, laterally more extensive dorsally than ventrally, excluded from intercaecal space and in asymmetrical patches about caeca in zone between cirrus-sac and posterior testis. Uterus short, intercaecal, constrained between posterior testis and genital pore, ventral to anterior testis; uterine seminal receptacle prominent in proximal part of uterus; metraterm muscular, well developed and occupy posterosinistral region of cirrus sac. Eggs oval, very few (5–17) (*n* = 20), 103–130 (118 ± 9.8) × 59–76 (68 ± 4.8). Excretory pore subterminal. Excretory vessel lateral loops span length of caeca.

Remarks: *Sagaratrema rajakarunae* is morphologically similar to *S. rajapaksei*, yet is much closer to *S. indicum* in COI mtDNA (Table 2 and Figure 2). Like in *S. rajapaksei*, in *S. rajakarunae* the distribution of the vitelline follicles is laterally extensive, differentiating it from *S. eugari* and *S. indicum*. However, in *S. rajakarunae*, the anterior extent of the vitelline follicles falls short of reaching the level of the ventral sucker by a small but consistent margin, whereas in *S. rajapaksei* the vitelline follicles always reach at least to the level of the ventral sucker and sometimes a little beyond anteriorly. Additionally, *S. rajakarunae* consistently has fewer eggs, 5–17 vs 23–47 in *S. rajapaksei*. A comparable gap between the vitelline follicles and ventral sucker is also characteristic of *S. eugari* and has been depicted for some material identified as *S. laticaudae*, specifically by Choe et al. (2020). Like *S. rajapaksei, S. rajakarunae* is distinguished from *S. laticaudae* by a relatively longer post-testicular zone, smaller testes and a smaller cirrus-sack.

*Sagaratrema rajakarunae* can be distinguished from *S. linguiforme* by shape of the posterior testis, larger eggs and smaller overall size.

### Phylogenetic results

**Figure 5.**
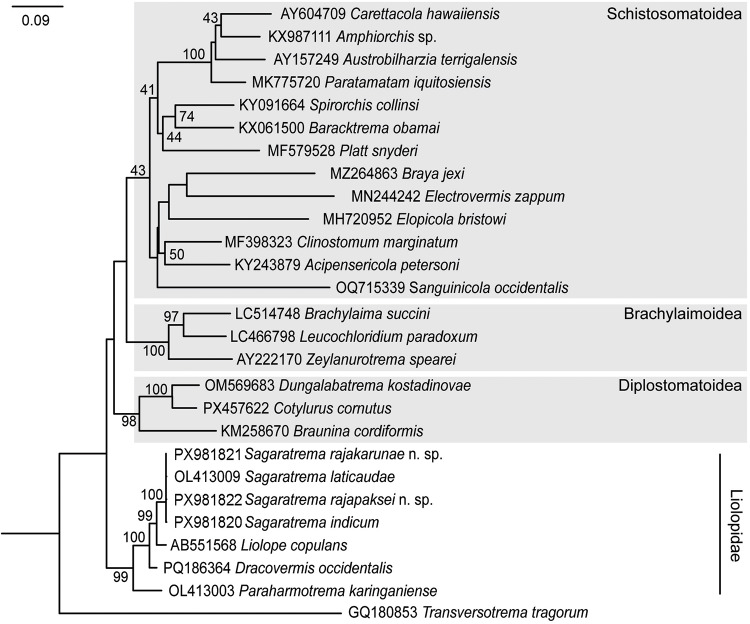
Hypothetical phylogenetic reconstruction for the Diplostomida based on maximum likelihood analysis of partial 28S rDNA. Bootstrap support less than 40 omitted. The scale bar indicates the expected number of substitutions per site. Figure 5 long description.

The phylogenetic hypothesis reconstructed for the Diplostomida via maximum likelihood analysis based on partial 28S recovered each of the Brachylaimoidea, Diplostomoidea and Schistosomatoidea as monophyletic (Figure 5). The Liolopidae was recovered as monophyletic and basal within the Diplostomida. Relationships between these major clades were poorly supported, but the topology is consistent with some recent previous analyses (e.g. Cutmore et al., 2023). Within the Liolopidae, species of *Sagaratrema* formed a strongly supported and derived clade, sister to the sequence for *Liolope copulans*.

## Discussion

### The marine liolopids: a return to the sea

Central to the proposal of *Sagaratrema* is the hypothesized ecological, host and biogeographic distinctions separating those species, parasites of mostly marine elapid snakes in the Indo-West Pacific, from the now monotypic *Harmotrema infecundum*, known only from a colubrid associated with freshwater in western Africa. These distinctions are interpreted here to reflect substantial separation in evolutionary history, and this is consistent with the morphological distinctions recognized here as the basis for the new genus concept.

We hypothesize that at least some species of *Sagaratrema* have entirely marine life cycles, that is, that they use a marine snail first intermediate host and marine fishes as second intermediate hosts, in addition to marine snakes as definitive hosts. Furthermore, we hypothesize that the origins of the Liolopidae were in freshwater, and that *Sagaratrema* is the only liolopid lineage that has subsequently invaded marine environments. Finally, this hypothesized narrative is framed as a return to the sea, because, although we suspect that the last common ancestral liolopid was a freshwater species, the origins of the Digenea are presumably marine (Cable, 1974; Cribb et al., 2001; Cribb et al., 2003).

The evidence supporting each of the components of the above evolutionary narrative is variously compelling. There are no records of first intermediate or second intermediate hosts for any species of *Sagaratrema*, and so the species examined here from Sri Lanka are hypothesized to be entirely marine based on the habits of the definitive hosts. Of the more than 70 extant species of substantially marine snakes (Rasmussen et al., 2011), the viviparous sea snakes (Elapidae: Hydrophinae: Hydrophinii) are the richest and most specialized for the marine environment. Although hydrophins can sometimes be found significant distances upstream in rivers (Rasmussen, 2001), most species typically have restricted activity ranges (Burns and Heatwole, 1998; Udyawer et al., 2015), and the snakes sampled here were procured from fishermen operating in inshore marine waters vs estuarine habitats. Therefore, we think the first intermediate hosts are marine snails found in proximity to the sampled snakes; the possibility of amphidromous or estuarine fishes transmitting infections from freshwater or estuarine snails to marine snakes is unlikely, because the parasites still require transport from the snakes back to the snail hosts.

**Figure 6.**
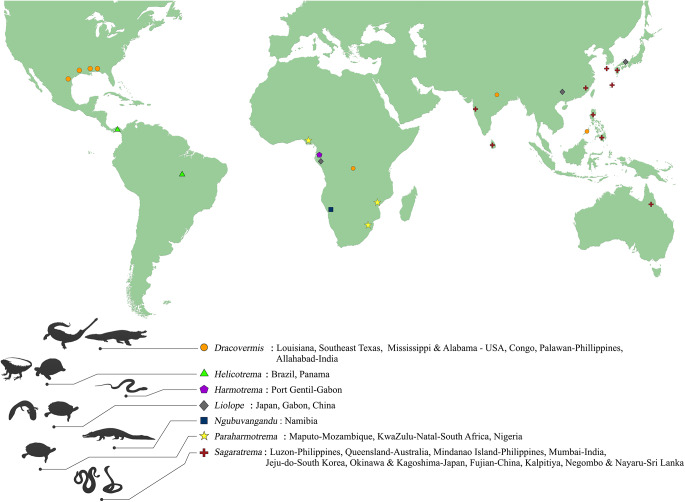
Global distribution of the 7 known genera within the family Liolopidae Dollfus, 1934. Figure 6 long description.

We predict a freshwater origin for the Liolopidae given that the earliest-branching lineages within the family have known or presumed freshwater life cycles (*Paraharmotrema* and then *Dracovermis*, see Figure 5 and Dutton et al., 2026), and that no liolopids other than *Sagaratrema* spp. are suspected to have marine life cycles. Additionally, the last common ancestor of the Liolopidae presumably predates the recent origins of marine elapids; the viviparous sea snakes and amphibious sea kraits transitioned independently from a terrestrial existence approximately 6–20 million years ago (Sanders et al., 2013; Lee et al., 2016; Heatwole et al., 2017; Kim et al., 2018; Sherratt et al., 2018). Although, time calibration of digenean phylogeny is extremely limited (see De Baets et al., 2015), the basal position and long branch separation of the Liolopidae within the Diplostomida, together with the broad geographic and definitive host range for the family (see Figure 6), suggests an origin comfortably older than for marine snakes. Indeed, Brooks and Overstreet (1978) hypothesized a Gondwanan last common ancestor for the Liolopidae no later than the Cretaceous.

Accepting a freshwater life cycle for the last common ancestral liolopid and that at least some species of *Sagaratrema* have marine life cycles, it is then either the case that ancestral species of *Sagaratrema* co-invaded the marine environment with their snake hosts or switched into marine snakes more recently. Both explanations are plausible. Regardless, our hypothesized evolutionary narrative requires that a host switch from freshwater to marine snails occurred, possibly with interim switches through estuarine snails. These first intermediate host switches could have been facilitated by the transitional period in the evolution of marine snakes from terrestrial ancestors (see Heatwole et al., 2017). However, it is alternatively possible that the parasites host-switched into a transitional snake species (whether ancestral or contemporary) and then subsequently into marine snails, such that it is not necessarily the case that the parasites co-invaded the marine environment with their snake hosts.

### The missing link?

The potentially important and enigmatic exception to the ecological and evolutionary narrative proposed here is *S. eugari*, the only species included in the new genus reported from snakes other than hydrophine elapids. Instead, it is known from the Philippine cobra, a terrestrial species, and the Southeast Asian bockadam, a semi-aquatic, mostly piscivorous and substantially marine species which also ranges through brackish, estuarine and freshwater habitats (Bernstein et al., 2024). Regardless, *S. eugari* is entirely consistent with *Sagaratrema* morphologically and shares none of the characters distinguishing *H. infecundum* from that concept, except, perhaps, that *S. eugari* is the smallest species of the new genus, comparable in size to *H. infecundum*.

Presuming that *S. eugari* indeed forms a natural group with the remaining species of *Sagaratrema*, the implications for our hypothesized evolutionary narrative depend on 2 pieces of information which are currently unknown: the phylogenetic position of *S. eugari*, basal or derived, relative to other species of *Sagaratrema*, and the specific ecology of the life cycle, that is, whether it exploits freshwater, estuarine or marine snails. Obtaining genetic and life cycle data for *S. eugari* and confirming its typical definitive hosts is therefore of particular interest and consequence.

### Richness, host-specificity and difficulty in species delineation

This study increases the number of liolopid species known from Indo-West Pacific marine snakes from 4 to 6 and is the first study to interpret a collection of such material as comprising more than a single species. The morphological and molecular distinctions recognized between species of *Sagaratrema* are slight. In particular, ITS2 rDNA, the most commonly used genetic marker for delineation of digenean species (Blasco-Costa, 2025), exhibited no variation among the 3 species from Sri Lanka. Critical to the delineation of these 3 species was that the 28S rDNA genotypes are similarly distinct in sympatry as each is relative to *S. laticaudae* from Japan, and that these genotypes were consistently corroborated by both COI mtDNA haplotypes and morphotypes.

Furthermore, our modest sampling of snakes found overlapping host and geographic ranges for the 3 species of *Sagaratrema* detected from Sri Lanka, including coinfections of *S. indicum* with both *S. rajapaksei* and *S. rajakarunae.* Thus, host-specificity is seemingly relaxed among hydrophins, and collections from even a single snake require scrutiny. Reliance on hologenophore specimens was therefore critical and any future investigation of novel *Sagaratrema* collections should use this approach to prospect for possible genetic diversity among morphologically conserved material.

The known richness of *Sagaratrema* is much lesser than for marine snakes (some 70 species, see Rasmussen et al., 2011). We think it likely that several more species of *Sagaratrema* are yet to be discovered, as marine snakes have been scarcely examined for helminths and the subtle morphological distinctions detected here allows the possibility that previous efforts unaided by genetic inference might have underestimated richness. However, we also think it unlikely that the richness of *Sagaratrema* will prove to match that of marine snakes, because many of the hydrophine elapids have specialized diets and so are seemingly unavailable hosts (Voris et al., 1978; Voris and Voris, 1983; Shine et al., 2004; Li et al., 2005; Brischoux and Lillywhite, 2011; De Silva et al., 2011).

### Contribution to the fauna of Sri Lanka

Together with *Tubulovesicula laticaudae* Parukhin, 1969 (Digenea: Hemiuridae) reported from this same investigation of marine snakes (Martin et al., 2023), the liolopids reported herein are the first parasites to be reported from marine snakes in Sri Lanka. Furthermore, *S. rajapaksei* and *S. rajakarunae* are the first new parasitic helminth species from marine wildlife in Sri Lanka to be proposed on the basis of an integrated taxonomic approach including inference from genetic data.

## Data Availability

Raw morphometric data are publicly and freely available at https://data.mendeley.com/datasets/33zjgbd3pc/1

## Figure 1 Long description

The map of Sri Lanka displays landing locations for marine snakes caught through fisheries bycatch. The map includes the Gulf of Mannar, Bay of Bengal and Laccadive Sea. Various symbols represent different snake species: Acrochordus granulatus is marked with a circle, Cerberus rynchops with a triangle, Hydrophis cyanocinctus with a diamond, Hydrophis curtus with a square, Hydrophis ornatus with a pentagon, Hydrophis schistosus with a hexagon and Hydrophis spiralis with a star. Locations include Baththalangunduwa, Kalpitiya, Negombo, Dehiwala and Nayaru. Each symbol is placed at specific landing sites, indicating where each species was found. The map provides a visual representation of the distribution of these marine snakes along the coastal waters of Sri Lanka.

Navigate back to Figure 1.

## Figure 2 Long description

The diagram is an unrooted neighbor-joining tree illustrating genetic relationships among species based on COI mitochondrial DNA and 28S ribosomal DNA. The tree is divided into two main sections, each representing different genetic data. On the left, sequences labeled as S. indicum are shown, with multiple branches indicating genetic variation within this species. The middle section features S. laticaudae, which is connected to S. indicum and S. rajakarunae, indicating genetic proximity. On the right, sequences labeled as S. rajakarunae and S. rajapaksei are displayed, with S. rajapaksei forming a distinct cluster. Dotted horizontal lines indicate which data were generated from each specimen. The scale bars at the bottom measure genetic distance in number of base positions, with the left bar representing a distance of 5.0 and the right bar representing 0.5.

Navigate back to Figure 2.

## Figure 3 Long description

The map illustrates the geographic distribution of Sagaratrema species in the Indo-West Pacific region. Specific locations are marked with symbols representing different species. In India, Mumbai is marked with a blue circle for S. indicum. Fujian, China and Queensland, Australia, are highlighted in green. In Sri Lanka, Kalpitiya, Negombo and Nayaru are marked with blue, red and yellow symbols for S. indicum, S. rajakapoori and S. rajalakshaei, respectively. In South Korea, Jeju is marked with a green square for S. usguri. In Japan, Kagoshima and Okinawa are marked with green squares for S. usguri. In the Philippines, Luzon and Mindanao Island are marked with orange triangles for S. linguliforme. The map uses different symbols to represent each species: blue circle for S. indicum, red triangle for S. linguliforme, green square for S. usguri, pink diamond for S. rajakapoori and yellow star for S. rajalakshaei.

Navigate back to Figure 3.

## Figure 4 Long description

The image contains five anatomical illustrations of parasites recovered from marine snakes in Sri Lanka. Illustrations A, B and C show whole-body ventral views of elongated, worm-like organisms with detailed internal structures. Illustration D is a close-up of terminal genitalia, with labels pointing to the cirrus-sac, ejaculatory duct, genital pore and uterus with eggs. Illustration E focuses on the ovarian complex, highlighting the ovary, Mehlis’ gland, uterine seminal receptacle and vitelline reservoir. These are black-and-white line drawings with stippling and shaded regions, accompanied by scale bars for reference. The figure serves a taxonomic and anatomical purpose, providing detailed views of the organisms' reproductive anatomy.

Navigate back to Figure 4.

## Figure 5 Long description

The phylogenetic tree is oriented vertically, depicting evolutionary relationships among Diplostomida based on maximum likelihood analysis of partial 28S rDNA. The tree begins at the top with the Schistosomatoidea clade, which includes several species such as Carcettacola hawaiiensis, Amphimerus sp. and Austrobilharzia terrigalensis, with bootstrap values of 41, 41 and 44 respectively. The Brachylaimoidea clade follows, containing species like Dicrocoelium sp. and Leucochloridium paradoxum, with bootstrap values of 100 and 100. Below this, the Diplostomoidea clade is shown, including species such as Cotylurus cornutus and Braunina cordiformis, with bootstrap values of 100 and 100. At the base of the tree, the Liolopidae clade is depicted, featuring species like Sagaratrema sp. and Paraharmostomum karinagiense, with bootstrap values of 100 and 100. The scale bar at the top left indicates the expected number of substitutions per site, set at 0.09. The tree illustrates the monophyletic nature of the Brachylaimoidea, Diplostomoidea and Schistosomatoidea clades, with Liolopidae being basal within the Diplostomida. Relationships between major clades are shown with varying levels of bootstrap support, indicating the strength of the phylogenetic hypothesis.

Navigate back to Figure 5.

## Figure 6 Long description

The map illustrates the global distribution of seven genera within the Liolopidae family, marked by distinct symbols. Each genus is represented by a unique symbol and color, with specific locations labeled. Dracovermis: Represented by an orange triangle, found in Louisiana, Southeast Texas, Mississippi & Alabama in the USA, Congo, Palawan in the Philippines and Allahabad in India. Holostroma: Indicated by a green circle, located in Brazil and Panama. Harmotrema: Shown by a purple square, found in Port Gentil, Gabon. Liolop: Marked by a yellow diamond, present in Japan and Gabu, China. Nugulobangus: Represented by a blue star, located in Japan. Paraharmotrema: Indicated by a red hexagon, found in Maputo, Mozambique, KwaZulu-Natal in South Africa and Nigeria. Sagaratrema: Shown by a brown pentagon, distributed across Luzon in the Philippines, Queensland in Australia, Mindanao Island in the Philippines, Mumbai in India, Jeju-do in South Korea, Okinawa & Kagoshima in Japan, Fujian in China, Kalpitiya, Negombo & Nuwara in Sri Lanka. The map provides a visual representation of the geographic spread of these genera, highlighting their presence in various regions across the world.

Navigate back to Figure 6.

## Table 1 Long description

The table lists representative taxa used for a 28S rDNA phylogenetic dataset, giving each taxon’s GenBank accession number and the publication source. Taxa are grouped by higher-level clades, including Liolopidae, Brachylaimoidea, Diplostomoidea, Schistosomatoidea, and Transversotrematoidea. Liolopidae includes Liolope copulans (AB551568) plus Dracovermis occidentalis (PQ186364), Paraharmotrema karinganiense (OL413003), and three Sagaratrema species with accessions PX981820 to PX981822 cited as “ex nobis.” Brachylaimoidea includes Brachylaima succini (LC514748), Leucochloridium paradoxum (LC466798), and Zeylanurotrema spearei (AY222170). Diplostomoidea includes Dungalabatrema kostadinovae (OM569683), Cotylurus cornutus (PX457622), and Braunina cordiformis (KM258670). Schistosomatoidea is the largest section, listing multiple families and accessions such as AY604709, KX987111, AY157249, MK775720, KY091664, KX061500, MF579528, MZ264863, MN244242, MH720952, MF398323, KY243879, and OQ715339. Transversotrematoidea is represented by Transversotrema tragorum (GQ180853). One taxon name includes a note that its GenBank registration uses a different name, so users should verify synonyms when retrieving sequences.

Navigate back to Table 1.

## Table 2 Long description

The table maps which Sagaratrema species were detected in different Hydrophis hosts sampled from two regions, the Bay of Bengal and the Gulf of Mannar, and lists the sample size for each host. In the Bay of Bengal, H. curtus had one sample and was positive only for S. rajapaksei. Bay of Bengal H. cyanocinctus had two samples and was positive for S. indicum and S. rajakarunae, but not S. rajapaksei. Bay of Bengal H. spiralis had one sample and was positive only for S. rajapaksei. In the Gulf of Mannar, H. schistosus had seven samples and was positive for S. indicum and S. rajapaksei, while Gulf of Mannar H. spiralis had one sample and was positive only for S. rajapaksei. Overall, S. rajapaksei is the most widespread across hosts and both regions, whereas S. indicum is limited to H. cyanocinctus and H. schistosus and S. rajakarunae is only recorded in H. cyanocinctus. Some host and parasite combinations overlap as coinfections, and several host groups have very small sample sizes, so absence in a cell may reflect limited sampling rather than true absence.

Navigate back to Table 2.

## Table 3 Long description

The table reports pairwise genetic differences among four Sagaratrema species, using partial 28S rDNA above the diagonal and partial COI mtDNA below the diagonal; COI intraspecific variation is listed on the diagonal. For 28S, differences are low across species, ranging from about 0.5 to 2.5 base positions. The largest 28S separation is between S. indicum and S. rajapaksei at about 1.5 to 2.5, while S. rajakarunae and S. laticaudae differ by about 1. For COI, S. indicum is much more divergent from S. rajapaksei at 41 to 42 than from S. rajakarunae at 8 to 9. COI differences between S. rajapaksei and S. rajakarunae are about 40. COI values involving S. laticaudae are not available, so comparisons for that species are limited to 28S only. COI within-species variation is small where reported, with 0 to 1 for S. indicum and S. rajapaksei and 0 for S. rajakarunae.

Navigate back to Table 3.

## Table 4 Long description

The table compiles published and new morphometric ranges for eight Sagaratrema taxa from Japan, Korea, the Philippines, China, India, and Sri Lanka, reporting body size and multiple organ measurements in micrometres. Overall body length varies widely, from about 1,900 to 4,000 in S. eugari up to about 8,000 to 8,800 in S. linguiforme; the Sri Lankan S. indicum and the two Sri Lankan species S. rajapaksei and S. rajakarunae span intermediate but broad ranges. Body width follows a similar pattern, with S. linguiforme among the widest and the Sri Lankan taxa generally narrower than the largest-bodied species. Sucker and pharynx dimensions are provided for most taxa; ventral sucker measurements are notably large in S. linguiforme compared with the others. Reproductive structures (cirrus-sac, testes, ovary) show substantial overlap among species, but S. linguiforme stands out with a much larger ovary than the other taxa. Egg size is relatively consistent across taxa, with lengths roughly around 100 to 148 and widths around 59 to 80, while reported egg counts vary from as low as 5 to 17 in S. rajakarunae to as high as 25 to 47 in S. rajapaksei, and some counts are taken from illustrations rather than measured directly. Proportional measures (such as forebody, cirrus-sac, posterior testis, and post-testis zone relative to body length) suggest the post-testis zone is especially large in S. eugari compared with the others. Several cells are missing, some values are derived from drawings, and specimen numbers differ by study, so comparisons should be treated as approximate rather than definitive.

Navigate back to Table 4.
